# Practice Patterns in Testing Procedures and Interpretation of Cardiopulmonary Exercise Testing

**DOI:** 10.1016/j.jacadv.2025.102479

**Published:** 2025-12-16

**Authors:** Timothy W. Churchill, Kristen Steinmetz, Anders Johnson, Daniel S. Kim, Jeffrey W. Christle, Jonathan Myers, Ankit B. Shah, Daniel R. Seifer, Nathaniel Moulson, Bradley J. Petek

**Affiliations:** a Division of Cardiology, Massachusetts General Hospital, Boston, Massachusetts, USA; b Cardiovascular Performance Program, Massachusetts General Hospital, Boston, Massachusetts, USA; c Cardiology Division, VA Palo Alto Health Care System, Division of Cardiovascular Medicine, Stanford University School of Medicine, Stanford University, Palo Alto, California, USA; d Division of Cardiology, University of Washington, Seattle, Washington, USA; e Sports & Performance Cardiology LLC, Chevy Chase, Maryland, USA; f Georgetown University School of Medicine, Washington, District of Columbia, USA; g Division of Pulmonary, Allergy, and Critical Care Medicine, Oregon Health & Science University, Portland, Oregon, USA; h Division of Cardiology and Sports Cardiology BC, University of British Columbia; i Sports Cardiology Program, Knight Cardiovascular Institute, Oregon Health & Science University, Portland, Oregon, USA

**Keywords:** athlete, cardiomyopathy, exercise, exercise testing, heart failure

**What is the clinical question being addressed?**

What are the practice patterns of testing procedures and interpretation standards for CPET laboratories?

**What is the main finding?**

There is significant heterogeneity in CPET protocols, supervision, interpretation standards, and further specialized testing. Clinical practice guidelines should address these differences in care.

Cardiopulmonary exercise testing (CPET) is increasingly utilized for diagnosis, prognosis, exercise prescription, and assessment of therapeutic responses among both healthy athletes and patients with cardiopulmonary symptoms or established cardiopulmonary disease.^1^ The primary aim of the current study was to assess practice patterns in clinical procedures and interpretation standards of CPET among testing laboratories.

We conducted a survey of CPET laboratories with the goal of defining practice patterns for testing procedures and clinical CPET interpretation. Inclusion criteria included health care providers or research scientists who worked in a clinical or research CPET laboratory. Duplicate responses from the same lab were removed. Surveys were available online in English only from February 1, 2025 to May 27, 2025 and were distributed to other known CPET laboratories by study investigators, through email to exercise physiologists registered on the American College of Sports Medicine Profinder online tool, and the survey was marketed at the 2025 American College of Sports Medicine Annual Meeting. Cardiology CPET laboratories were defined as those primarily caring for patients with myocardial disease/congenital heart disease, sports cardiology patients, and general CPET laboratories as those caring for broader-range cardiac/pulmonary patient populations. The Mass General Brigham Institutional Review Board approved this study. All participants gave informed consent. Responses were presented using standard descriptive statistics. Statistical analyses were performed using R (version 4.4.2).

A total of 72 CPET laboratories from 58 hospital systems completed the survey (70 United States, 1 Canada, 1 Australia). The primary patient population for CPET laboratories included patients with myocardial disease/congenital heart disease (36/72, 50%), followed by sports cardiology patients (14/72, 19%), athletes seeking exercise recommendations (9/72, 13%), other populations (6/72, 8%, including cancer survivors (2), Parkinson disease, executive health programs, preterm birth, students), multiple cardiac/pulmonary patient populations (4/72, 6%), and pulmonary disease patients (3/72, 4%). The most common equipment available for CPET testing included treadmills (68/72, 94%), followed by upright cycle ergometers (52/72, 72%), recumbent cycle ergometers (14/72, 19%), and supine cycle ergometers (8/72, 11%). An overview of CPET procedures and interpretation standards is presented in Table 1.

**Table 1:** Cardiopulmonary Exercise Testing Procedures and Interpretation Standards

	All CPET Laboratories (n = 72)	Cardiology CPET Laboratories (n = 54)
Where is the supervising provider during testing?		
In close vicinity (eg, on the same floor)	37 (51)	32 (59)
In the exam room during testing	14 (19)	11 (20)
In the same building but on a different floor	9 (13)	7 (13)
No clinical supervision	6 (8)	0 (0)
On the medical campus but different building or remote (available for contact)	6 (8)	4 (7)
What exercise equipment is available?^a^		
Treadmill	68 (94)	52 (96)
Upright cycle ergometer	52 (72)	40 (74)
Recumbent cycle ergometer	14 (19)	13 (24)
Supine cycle ergometer	8 (11)	8 (15)
Arm crank ergometer	3 (4)	2 (4)
Other^b^	3 (4)	3 (6)
Rowing ergometer	2 (3)	2 (4)
Primary treadmill protocol^c^		
Bruce protocol	25 (37)	18 (35)
Customized ramp protocol (individualizing the speed of the treadmill based on the patient)	25 (37)	21 (41)
Other^b^	9 (13)	5 (10)
Modified Bruce protocol	4 (6)	3 (6)
Naughton protocol	2 (4)	2 (4)
Modified Naughton protocol	1 (2)	1 (2)
Balke protocol	1 (2)	1 (2)
Normative values for peak $\dot{V}$O_2_—cycle ergometer^c^		
Wasserman	25 (53)	19 (54)
FRIEND Registry	13 (28)	9 (26)
Other^b^	7 (15)	5 (14)
Jones	2 (4)	2 (6)
Normative values for peak $\dot{V}$O_2_—treadmill^c^		
Wasserman	25 (56)	20 (57)
FRIEND Registry	12 (27)	8 (23)
Other^b^	5 (11)	4 (11)
Jones	3 (7)	3 (9)
Invasive CPET performed?	12 (17)	12 (22)
CPET with simultaneous echocardiography performed?	26 (36)	24 (44)

ACSM = American College of Sports Medicine; CPET = cardiopulmonary exercise testing; FRIEND = Fitness Registry and the Importance of Exercise National Database; NDKS = Nustad Dressler Kobes Saghiv; YMCA = Yong Men’s Christian Association.

a Multiple answers allowed (choose all that apply).

b Other exercise equipment (both all CPET laboratories and cardiology CPET laboratories- 1/3 SciFit total body trainer, 1/3 swim flume, 1/3 NuStep recumbent cross trainer), other treadmill protocols (all CPET laboratories- 6/9 internally created custom protocols, 1/9 Cornell, 1/9 Gerkin, 1/9 NDKS, cardiology CPET laboratories- 4/5 internally created custom protocols, 1/5 Cornell), other normative values for peak $\dot{V}$O_2_—cycle ergometer (all CPET laboratories- 1 ACSM, 1 Astrand, 1 Wasserman + FRIEND, 1 Burns 2021, 1 Mayo Clinic normative local values, 1 YMCA, 1 Bruce 1973 + Drinkwater 1975, cardiology CPET laboratories- 1 ACSM, 1 Wasserman + FRIEND, 1 Burns 2021, 1 Mayo Clinic normative, 1 Bruce 1973 + Drinkwater 1975), other normative values for peak $\dot{V}$O_2_—treadmill (all CPET laboratories 1 ACSM, 1 Astrand, 1 Wasserman + FRIEND, 1 Mayo Clinic normative local values, 1 Bruce 1973 + Drinkwater 1975, cardiology CPET laboratories).

c Partial data available. Primary treadmill protocol (all CPET laboratories n = 67, cardiology CPET laboratories n = 51), normative values for peak $\dot{V}$O_2_—cycle ergometer (all CPET laboratories n = 47, cardiology CPET laboratories n = 35), normative values for peak $\dot{V}$O_2_—treadmill (all CPET laboratories n = 45, cardiology CPET laboratories n = 35).

Among cardiology CPET laboratories (n = 54, from 45 institutions), the supervising provider is in close vicinity (on the same floor) in 32/54 (59%) laboratories, in the testing room in 11/54 (20%), in the same building on a different floor in 7/54 (13%), and on the medical campus in a different building or remote and available for contact in 4/54 (7%). Cardiology CPET laboratories most commonly utilize customized ramp protocols (21/51, 41%) for treadmill testing, followed by the Bruce protocol (18/51, 35%) and other protocols (5/51, 10%, including 4/5 internally created other custom/standardized protocols and 1/5 Cornell protocol). Among cardiology CPET laboratories not primarily focused on sports cardiology, the most common treadmill protocol is the Bruce protocol (16/38, 42%). A total of 24/45 (53%) institutions with cardiology CPET laboratories perform simultaneous CPET and echocardiography (CPET-Echo) and 12/45 (27%) perform invasive CPET. For the institutions performing invasive CPET (with pulmonary artery and/or radial arterial catheters), 6/12 (50%) perform testing on upright and 6/12 (50%) on supine cycle ergometers.

The most utilized normative values for peak oxygen consumption ($\dot{V}$O_2-PEAK_) for the cycle ergometer are the Wasserman equations (25/47, 53%), followed by the FRIEND (Fitness Registry and the Importance of Exercise National Database) Registry (13/47, 28%) and various other equations (9/47, 19%, Table 1). Similarly, the most common normative values for $\dot{V}$ O_2-PEAK_ for the treadmill are the Wasserman equations (25/45, 56%), followed by the FRIEND Registry (12/45, 27%) and various other equations (8/45, 18%, Table 1).

Among CPET laboratories that test athletes (sports cardiology patients or exercise performance laboratories), 17/22 (77%) use $\dot{V}$O_2-PEAK_ reference equations derived from the general population, and 16/25 (64%) commonly perform customized extra testing to recreate symptoms (eg, sprint testing in a track athlete with palpitations) in addition to a traditional maximal effort CPET.

This study assessed clinical procedures and interpretation standards across CPET testing laboratories with the following key findings. First, there is significant heterogeneity in exercise testing protocols, testing supervision, and reference equations for $\dot{V}$O_2-PEAK_ among CPET laboratories. This heterogeneity in testing protocols and interpretation may lead to significant variation in testing results between different CPET laboratories, so any test that changes clinical management should be carefully scrutinized to understand the testing conditions and interpretation standards. Second, among institutions with cardiology CPET laboratories, only 53% offer CPET-Echo and 27% invasive CPET testing. Both CPET-Echo and invasive CPET may improve the diagnostic accuracy in conditions such as unexplained dyspnea, heart failure, and complex cardiovascular physiology (eg, pulmonary hypertension), and broader dissemination of these testing approaches may improve the care of patients with these conditions.^2^^,^^3^ Finally, athletes frequently undergo CPET in the setting of exertional symptoms or to differentiate adaptive exercise-induced cardiovascular remodeling vs pathologic cardiovascular disease, but 77% of CPET laboratories that care for athletes use $\dot{V}$O_2-PEAK_ equations that are not specific for athletes. In addition, the use of customized exercise provocation to attempt to recreate athletes’ symptoms was variable, noted in <2/3 of laboratories. Previous studies have demonstrated that customized exercise testing in athletes may increase diagnostic yield by ∼40%, so further education is needed among CPET laboratories caring for symptomatic athletes to increase uptake of this important practice.^4^ Notably, there are no athlete-specific clinical practice statements on CPET interpretation, the development of which may offer an opportunity for more uniform guidance in this population.

This study has several limitations. First, this survey was completed by any health care providers (exercise physiologist, physician, advanced practice provider) or research scientists who work in a CPET lab, so responses may vary by provider type. Second, due to survey distribution methods, the response rate cannot be accurately calculated. Third, the survey was completed primarily by CPET laboratories based in the United States (97%) and laboratories that care for cardiology patients (75%), so findings may not generalize to other countries or laboratories focusing on other clinical populations. Finally, there is likely inherent bias in survey response given the methods employed, and the number and characteristics of nonresponders could not be evaluated. Nevertheless, these data demonstrate significant variation in practice patterns in CPET protocols and interpretation, raising opportunities for standardization and quality improvement.

## Funding support and author disclosures

Dr Churchill has received funding from the National Institute of Health/10.13039/100000050National Heart, Lung, and Blood Institute (5K23HL159262-03) and has received compensation for his work with the Boston Bruins organization. Dr Shah has received compensation for his work as sports cardiology consultant from MedStar Health and the U.S. Olympic and Paralympic Committee. Dr Kim is supported by the Wu-Tsai Human Performance Alliance as a Clinician-Scientist Fellow, the Stanford Center for Digital Health as a Digital Health Scholar, the Pilot Grant from the Stanford Center for Digital Health, NIH 1L30HL170306, the Robert A. Winn Excellence in Clinical Trials Career Development Award, the American Heart Association (AHA) Career Development Award (AHA 25CDA1436622), and the American Diabetes Association (ADA) Pathway to Stop Diabetes Initiator Award (7-25-INI-11). Dr Petek’s Sports Cardiology Program has received compensation for preparticipation cardiovascular screening for the National Hockey League Scouting Combine, University of Portland, and Portland State University. Additionally, his Sports Cardiology Program has received funding from the Joel Cornette Foundation for the Outcomes Registry for Cardiac Conditions in Athletes study. All other authors have reported that they have no relationships relevant to the contents of this paper to disclose.
